# Restoration of the nasopharyngeal response after bilateral sectioning of the anterior ethmoidal nerve in the rat

**DOI:** 10.14814/phy2.13830

**Published:** 2018-08-14

**Authors:** Paul F. McCulloch, Karyn M. DiNovo

**Affiliations:** ^1^ Department of Physiology Midwestern University Downers Grove Illinois

**Keywords:** Anterior ethmoidal nerve, Fos, medullary dorsal horn, nasal stimulation, nasopharyngeal response

## Abstract

In response to stimulation of the nasal passages with volatile ammonia vapors, the nasopharyngeal reflex produces parasympathetically mediated bradycardia, sympathetically mediated increased peripheral vascular tone, and apnea. The anterior ethmoidal nerve (AEN), which innervates the anterior nasal mucosa, is thought to be primarily responsible for providing the sensory afferent signals that initiate these protective reflexes, as bilateral sectioning causes an attenuation of this response. However, recent evidence has shown cardiovascular responses to nasal stimulation with ammonia vapors are fully intact 9 days after bilateral AEN sectioning, and are similar to control animals without bilaterally sectioned AENs. To investigate this restoration of the nasopharyngeal response, we recorded the cardiorespiratory responses to nasal stimulation with ammonia vapors immediately after, and 3 and 9 days after, bilateral AEN sectioning. We also processed brainstem tissue for Fos to determine how the restoration of the nasopharyngeal response would affect the activity of neurons in the medullary dorsal horn (MDH), the part of the ventral spinal trigeminal nucleus caudalis region that receives primary afferent signals from the nose and nasal passages. We found 3 days after bilateral AEN sectioning the cardiorespiratory responses to nasal stimulation are partially restored. The bradycardic response to nasal stimulation is significantly more intense 3 days after AEN sectioning compared to Acute AEN sectioning. Surprisingly, 3 days after AEN sectioning the number of Fos‐positive neurons within MDH decreased, even though the cardiorespiratory responses to nasal stimulation intensified. Collectively these findings indicate that, besides the AEN, there are alternate sensory pathways that can activate neurons within the trigeminal nucleus in response to nasal stimulation. The findings further suggest trigeminal neuronal plasticity involving these alternate sensory pathways occurs in as few as 3 days after bilateral AEN sectioning. Finally, activation of even a significantly reduced number of MDH neurons is sufficient to initiate the nasopharyngeal response.

## Introduction

All mammals, including humans, have an autonomic reflex that facilitates survival during underwater submergence. This so‐called diving response includes a parasympathetically mediated bradycardia, a sympathetically mediated increase in peripheral vascular tone, and apnea (Butler and Jones 1997; Panneton 2013). A related reflex, the nasopharyngeal response, produces these same efferent responses in anesthetized animals upon stimulation of the nasal passages (McCulloch 2012). This reflex is primarily a defensive reflex protecting the airways and lungs against noxious chemical irritants (Panneton 2013). Primary afferent fibers innervating the nose and nasal passages are important for initiating both the diving and nasopharyngeal responses (McCulloch 2012; Panneton 2013). The anterior ethmoidal nerve (AEN), which innervates the anterior nasal mucosa (Greene 1963), is thought to be primarily responsible for providing the sensory afferent signals that initiate these protective reflexes (McCulloch 2012; Panneton 2013). For instance, electrical stimulation of the AEN produces the same intense cardiorespiratory reflex as does the stimulation of the nasal passages (Dutschmann and Herbert 1998; McCulloch et al. 1999; Rozloznik et al. 2009). Additionally, bilateral sectioning of the AENs causes an attenuation of the nasopharyngeal response (Rybka and McCulloch 2006).

More recently, however, the importance of the AEN in initiating these reflexes has been placed in doubt. Rats voluntarily diving through an underwater maze were able to initiate a full diving response, including an intense bradycardia and an increase in arterial blood pressure, after the AENs were cut bilaterally (Chotiyanonta et al. 2013). Further, when these same rats were anesthetized, the cardiorespiratory responses to nasal stimulation with ammonia vapors were fully intact 9 days after AEN sectioning, and were similar to control animals without bilaterally sectioned AENs (Chotiyanonta et al. 2013). Thus there is a discrepancy between the results from Rybka and McCulloch (2006) and Chotiyanonta et al. (2013). In anesthetized rats with bilateral AEN sectioning the nasopharyngeal response is attenuated acutely (Rybka and McCulloch 2006), but returns fully 9 days after AEN sectioning (Chotiyanonta et al. 2013).

The present experiments were therefore designed to investigate the restoration of the nasopharyngeal response after bilateral sectioning of the AENs. We recorded the cardiorespiratory responses to nasal stimulation with ammonia vapors 3 and 9 days after bilateral AEN sectioning. We also processed brainstem tissue for Fos, a measure of neuronal activation (Dragunow and Faull 1989; Hughes and Dragunow 1995; Coggeshall 2005), to determine how the restoration of the nasopharyngeal response would affect activity of neurons in the medullary dorsal horn (MDH), the part of the ventral spinal trigeminal nucleus caudalis region that receives primary afferent signals from the nose and nasal passages (Anton and Peppel 1991; Hollandsworth et al. 2009).

## Materials and Methods

All study procedures were approved by the Midwestern University IACUC. Adult male Sprague‐Dawley rats were purchased from a commercial vendor (Harlan, Indianapolis IN) and were housed within the Midwestern University Animal Facility. Rats were initially caged in pairs and were housed in accordance with NIH guidelines for the care and use of laboratory animals. Food and water were available ad libitum. After bilateral AEN surgery (see below), rats were caged individually.

Experiments were designed to determine the effect bilateral AEN sectioning, and recovery from bilateral AEN sectioning, has upon the responses to nasal stimulation with ammonia vapors. In rats with bilateral AEN sectioning, a 0 (Acute), 3, or 9 day delay separated the AEN surgery from the testing of the responses to nasal stimulation. These responses were compared to the responses from Control rats that had Sham AEN sectioning. In all rats receiving repetitive nasal stimulation, the cardiorespiratory responses were recorded and then later the ventral spinal trigeminal nucleus caudalis region was inspected for the presence of Fos‐positive neurons.

### AEN surgery

For bilateral AEN sectioning, rats were initially anesthetized using isoflurane (5%/vol initial induction, then 2–3%/vol maintenance, in 95% O_2_ and 5% CO_2_) and then placed in a stereotaxic device (Kopf Instruments, Tujunga CA). Under aseptic conditions, the orbit was exposed and the eyeball was retracted laterally. Using a stereoscopic surgical microscope, the AEN was identified within the orbit as it passed through the anterior ethmoidal foramen. The AEN was separated from the accompanying artery and vein. In all animals that received AEN sectioning, a 1 mm piece of nerve was removed from the AEN, which confirmed the denervation. In Sham animals, the AEN was isolated but left intact. Surgical procedures were repeated contralaterally. Ketoprofen (3–5 mg/kg sc) was used as a postsurgical analgesic for animals recovering from AEN surgery (3 and 9 day rats) both immediately after the surgery and again 24 h later.

### Nasal stimulation with ammonia vapors

The nasal passages of rats (*N *= 20; *n *= 5 in each of four groups; weights: 270–370 g) were stimulated using methods similar to those of Rybka and McCulloch (2006). In Acute and Sham rats, still anesthetized with isofluorane from the AEN surgery, the right femoral vein and artery were cannulated for administration of drugs and recording of blood pressure, respectively. The trachea was transected and a tube was inserted caudally to enable breathing, and a nasopharyngeal tube was inserted rostrally to enable stimulation of the nasal passages (see below). Other rats, after a 3 or 9 day delay following bilateral AEN sectioning, were re‐anesthetized with isoflurane (5%/vol initial induction, then 2–3%/vol maintenance, in 95% O_2_ and 5% CO_2_) and received the same cannulations. After completion of all surgical procedures, all rats were gradually switched to urethane anesthesia (1 g/kg, iv) and breathed room air through the caudal‐facing tracheal cannula.

For nasal stimulation trials, a cotton swab soaked in 100% ammonia was held close to the external nares for 5 s while a suction pump connected to the nasopharyngeal tube drew the ammonia vapors through the nasal passages. The nasal passages of rats were stimulated every 5 min for 2 h, for a total of 24 stimulation trials. To record blood pressure, the arterial cannula was connected to a pressure transducer. To record respiration, a temperature probe was placed in the tracheal cannula. The pretrial parameters were measured within 30 sec of the trial, during a 10 sec period when all the parameters were in steady state after turning on the nasopharyngeal suction pump. For the trial, the time period when obvious cardiorespiratory changes were occurring determined the duration of the response and thus when trial cardiorespiratory measurements were taken. All electronic signals were amplified (Grass 7P122; Astro‐Med, West Warwick RI) and transmitted through an A/D board (micro1402; CED, Cambridge UK) to a computer where they were stored and later analyzed using appropriate software (Spike2; CED). Heart rate was determined from pulse pressure intervals.

### Fos‐positive neurons within the spinal trigeminal nucleus

One hour after the completion of all nasal stimulation trials, each rat was deeply anesthetized with a concentrated sodium pentobarbital solution (Sleepaway, iv; Fort Dodge IA) and perfused transcardially with 0.9% saline followed by 4% paraformaldehyde in 0.1 mol/L phosphate buffered saline. The brain and rostral spinal cord were removed and placed in 20% phosphate‐buffered sucrose solution and refrigerated for at least 24 h at 4°C. The brainstem was then cut in transverse frozen sections (50 *μ*m) from the rostral spinal cord to the inferior colliculus. A 1 in 3 series was immunologically processed for Fos. The Fos staining procedure consisted of incubating sections at room temperature overnight in a primary antibody (rabbit polyclonal IgG specific for c‐fos p62, 1:1000 dilution; SC‐052, Santa Cruz, Dallas TX) and then incubating them in a secondary antibody (goat anti‐rabbit biotinylated secondary IgG, 1:200 dilution; Vector, Burlingame CA) for 1 h. The sections were then washed in Vectastain Elite solution (Vector) for 1 h for signal amplification. DAB (0.05%) enhanced with nickel was used as the chromagen to visualize the Fos immunoreactive product. This immunohistological procedure produced a black nucleus in Fos‐positive neurons. The sections were serially mounted on gelatin‐coated glass slides, counterstained with Neutral red (0.03% at pH 4.45), dehydrated in alcohol, defatted in xylene, and coverslipped. Slides were re‐identified with a random code to blind the identity of the rat during counting of Fos‐positive neurons. This blinding code was not broken until the completion of all neuronal counting.

With the aid of a brain atlas (Paxinos and Watson 1998), Fos‐positive neurons were counted bilaterally in the ventral spinal trigeminal nucleus caudalis region, using a Nikon E600 light microscope and Northern Eclipse software (Empix Imaging; Mississauga ON). Fos‐positive neurons were darker in intensity than other nearby neurons. The spinal trigeminal caudalis region was located between the pyramidal tract caudally and obex rostrally (9–10 sections), and included the superficial MDH and the scattered neurons of the paratrigeminal nucleus located within the adjacent spinal trigeminal tract. Fos‐positive neurons were counted in the ventral half of the MDH superficial laminae (laminae I and II). Fos‐positive neurons were also counted in the ventral half of the paratrigeminal nucleus, near the ventral pole of MDH.

### Data presentation and statistical analysis

All cardiorespiratory and Fos data were compiled in Excel. Original cardiorespiratory traces were analyzed using Spike2. Heart rate (HR, beats/min (bpm)), mean arterial blood pressure (MAP, mmHg), and respiratory rate (RR, breaths/min) are presented as mean ± standard error (SE). The duration of apnea, if present, was also measured during the period of nasal stimulation. The number of Fos‐positive neurons is presented as the mean number of Fos‐positive neurons per hemisection ± SE appearing in each brain region. Data were tested for statistical significance using two‐way and one‐way ANOVAs, and *t*‐tests, as necessary (SigmaPlot 12; Systat, San Jose CA), with significance set at *P* < 0.05. For two‐way ANOVAs, the main effects were Time (Sham, Acute, 3 Day, and 9 Day) and Trial (pre and during ammonia vapor trials). In the case of a significant *P*‐value, post hoc testing with the Holm‐Sidak Pairwise Multiple Comparison Procedure determined significantly different groups. In some two‐way ANOVAs, the normality test failed, in which case a repeated measures two‐way ANOVA was performed. All graphs were created using SigmaPlot. Brain images were taken using a digital camera (Micropublisher; Q‐Imaging, Surrey BC) attached to the Nikon microscope, and Northern Eclipse software. Figures were composed and labeled using CorelDRAW (Corel, Ottawa ON).

To facilitate comparisons of Fos labeling patterns between different groups, sections were organized into six caudal‐rostral levels, each approximately 200 *μ*m thick, from the pyramidal decussation caudally to obex rostrally. Data (Fos‐positive neurons) from all individual brainstem sections were superimposed on the appropriate cross‐section, providing representative labeling at each of the six caudal‐rostral levels. The pyramidal decussation represented the transition between the rostral spinal cord and the caudal medulla. Calamus scriptorius was defined as the most caudal extent of area postrema, just as the dorsal columns separate. Obex was defined as the opening of the central canal into the fourth ventricle.

## Results

### Cardiorespiratory

**Figure 1:**
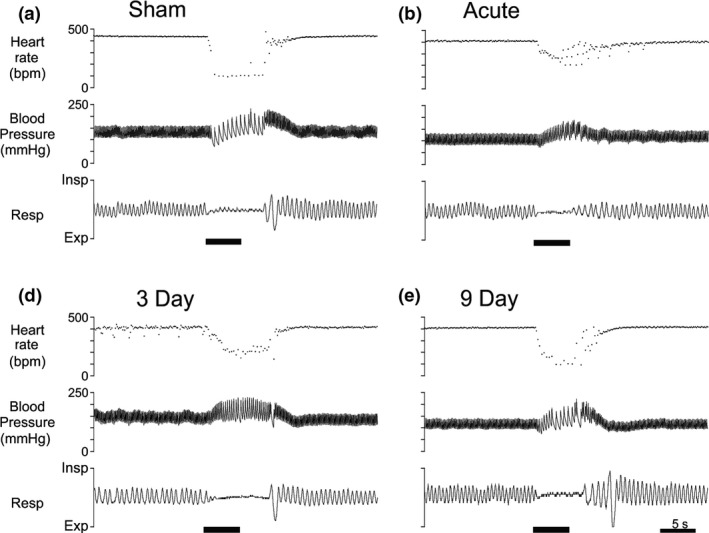
Cardiorespiratory responses to repetitive stimulation of the nasal passages with ammonia vapors in rats with AENs intact (A, Sham) and with AENs cut bilaterally (B, Acute; C, 3 Day; D, 9 Day). Sham received nasal stimulation immediately after sham AEN surgery. Acute received nasal stimulation immediately after bilateral AEN sectioning. Other rats experienced a 3 or 9 day delay between bilateral AEN sectioning and nasal stimulation. In each panel, from top: heart rate, pulsatile arterial blood pressure, and respiration (Resp; up is inspiration (Insp) and down is expiration (Exp). Solid bar underneath each tracing indicates period of nasal stimulation.

In Sham rats with intact AENs (Fig. 1A; Table 1), ammonia vapor stimulation of the nasal passages caused a significant 53% decrease in HR (from 365 ± 10 to 172 ± 18 beats/min, with the lowest HR reaching 83 ± 13 beats/min), a significant 16% increase in MAP (from 122 ± 6 to 142 ± 4 mmHg), a significant 82% decrease in respiratory rate (from 98 ± 2 to 18 ± 2 breaths/min), and apnea lasting 9.1 ± 1.3 sec.

**Table 1:** Heart Rate (HR; beats/min), mean arterial Blood Pressure (MAP; mmHg), respiratory rate (RR; breaths/min), and apnea (s) responses before (pretrial) and during (trial) 5 sec of nasal stimulation with ammonia vapors

	Sham	Acute	3 Days	9 Days
Pretrial HR	365 ± 10	392 ± 26	364 ± 10	403 ± 11
Trial HR	172 ± 18	318 ± 53	227 ± 35	232 ± 22
Lowest HR	83 ± 13	239 ± 63	126 ± 37	117 ± 17
Pretrial MAP	122 ± 6	125 ± 2	130 ± 2	135 ± 3
Trial MAP	142 ± 4	153 ± 3	148 ± 5	155 ± 2
Pretrial RR	98 ± 2	107 ± 8	95 ± 7	103 ± 5
Trial RR	18 ± 2	64 ± 20	31 ± 10	27 ± 4
Apnea	9.1 ± 1.3	3.3 ± 1.3	6.7 ± 1.3	5.3 ± 0.3

The lowest HR and apnea duration were determined by the longest pulse pressure interval and longest duration without respiratory effort, respectively, during the nasal stimulation trial. The anterior ethmoidal nerve (AEN) was sectioned bilaterally in all animals except in Sham rats. Sham received nasal stimulation immediately after sham AEN sectioning. Acute received nasal stimulation immediately after bilateral AEN sectioning. Other rats experienced a 3 or 9 day delay between bilateral AEN sectioning and nasal stimulation. *N *= 5 for each of the four groups.

**Figure 2:**
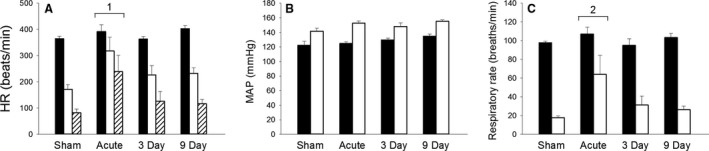
Mean Heart Rate (HR), arterial Blood Pressure (MAP), and respiratory rate responses (±SE) during nasal stimulation with ammonia vapors in rats with AENs intact (Sham) and with AENs cut bilaterally (Acute, 3 Day and 9 Day). Solid bar: pretrial; Open bar: trial; Cross‐hatched bar: Lowest heart rate during the trial. Nasal stimulation causes (A) a significant decrease in heart rate; (B) a significant increase in mean arterial blood pressure; and (C) a significant decrease in respiratory rate. Using two‐way ANOVAs: 1* *= HR responses to nasal stimulation significantly different in Acute compared with Sham, 3 and 9 day rats; 2* *= trial respiratory rate was significantly greater in Acute than in Sham. *N* = 5 for each of the four groups.

After sectioning the AENs bilaterally (Fig. 1), the HR responses to ammonia vapor stimulation of the nasal passages were attenuated compared to Sham (Fig. 2A; Table 1). The greatest attenuation of the bradycardia occurred in Acute rats. However, after a delay of 3 or 9 days between AEN sectioning and nasal stimulation, the bradycardia intensified compared to the response from Acute rats. A two‐way ANOVA post hoc analysis indicated HR responses to nasal stimulation were significantly different in Acute compared with Sham, 3 and 9 day rats (*P* < 0.001; Fig. 2A). The absolute decrease in HR (pretrial HR minus trial HR) was 192 ± 14 beats/min in the Sham rats, 74 ± 31 beats/min in the Acute rats, and 137 ± 32 and 171 ± 28 beats/min in the 3 and 9 day rats, respectively. A one‐way ANOVA indicated the absolute decrease in HR was significantly different in Sham versus Acute (*P *= 0.037). However, one‐way ANOVAs indicated the trial HR (*P *= 0.061) and the lowest HR (*P *= 0.051) were not significantly different between the four groups.

After sectioning the AENs bilaterally, the blood pressure responses to ammonia vapor stimulation of the nasal passages were both qualitatively and quantitatively similar to Sham (Fig. 2B; Table 1). A two‐way ANOVA indicated nasal stimulation caused a significant increase in MAP in all four groups (*P* < 0.001), while one‐way ANOVAs indicated pretrial MAP (*P *= 0.088) and trial MAP (*P *= 0.092) were not significantly different between the four groups. After sectioning the AENs bilaterally, the respiratory responses to ammonia vapor stimulation of the nasal passages were attenuated compared to Sham (Fig. 2C; Table 1). A two‐way ANOVA indicated nasal stimulation caused a significant decrease in respiratory rate in all four groups (*P* < 0.001; Fig. 2C); post hoc analysis indicated a trial respiratory rate significantly greater in Acute than in Sham (*P *= 0.026). A one‐way ANOVA indicated apnea was significantly shorter in Acute than in Sham (Table 1; *P *= 0.018).

### Fos‐positive neurons

Fos‐positive neurons were counted bilaterally within the ventral MDH and ventral paratrigeminal nucleus. Other brainstem locations contained Fos‐positive neurons, but these areas were not quantified.

**Figure 3:**
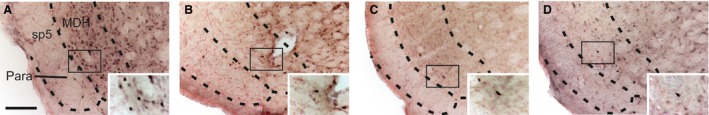
Brightfield photomicrographs of Fos‐positive neurons within the ventral trigeminal region at the level of calamus scriptorius after repetitive nasal stimulation with ammonia vapors in rats with AENs intact (A, Sham) and with AENs cut bilaterally (B, Acute; C, 3 Day; and D, 9 Day). Fos‐positive neurons were present in the ventral tip of the superficial laminae of the medullary dorsal horn (MDH), as well as in the ventral paratrigeminal nuclei (Para) located within the spinal trigeminal tract (sp5). Scale bar in A is 100 *μ*m for panels A‐D. Insets are examples of Fos‐positive neurons at 100x magnification of indicated rectangular region. All four panels correspond to Figure 77 from Paxinos and Watson (1998).

**Figure 4:**
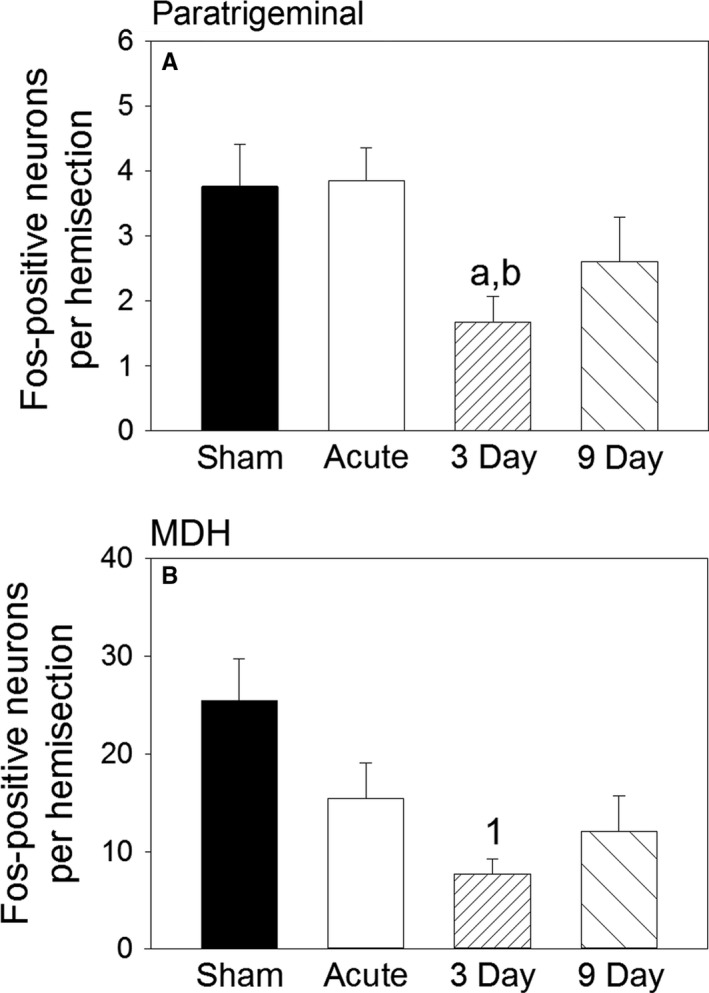
Total Fos‐positive neuron counts (mean ± SE) within the ventral (A) paratrigeminal nucleus and (B) MDH after repetitive nasal stimulation with ammonia vapors. Sham received nasal stimulation immediately after sham AEN sectioning. Acute received nasal stimulation immediately after bilateral AEN sectioning. Other rats experienced a 3 or 9 day delay between bilateral AEN sectioning and nasal stimulation. Using one‐way ANOVA: 1* *= significantly different from Sham. Using *t*‐tests: a* *= significantly different from Sham; b* *= significantly different from Acute. *N *= 5 for each of the four groups.

**Figure 5:**
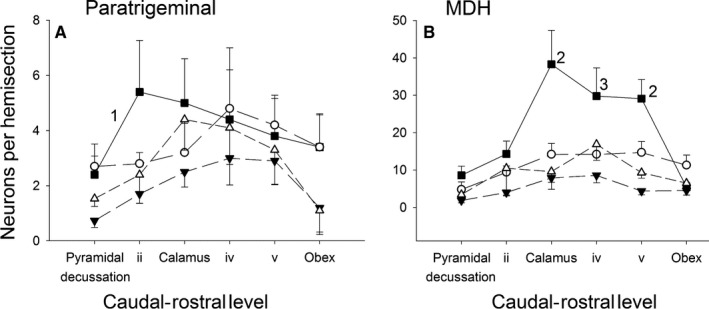
Counts of Fos‐positive neurons (mean ± SE) within the ventral (A) paratrigeminal nucleus and (B) medullary dorsal horn at each of six caudal‐rostral levels. Filled square (▴; *N *= 5): Sham; Open circle (○; *N *= 5): Acute; Filled down triangle (▾; *N *= 5): 3 Day; and Open up triangle (▵; *N *= 5): 9 Day. Using two‐way ANOVA: 1* *= Sham is significantly different from 3 Day. Using one‐way ANOVAs at individual caudal‐rostral levels: 2* *= significantly different from other three groups; 3* *= significantly different from 3 Day.

In Sham rats with intact AENs, ammonia vapor stimulation of the nasal passages caused activation of 3.8 ± 0.6 and 25.5 ± 4.3 Fos‐positive neurons per hemisection within the ventral paratrigeminal nucleus and ventral MDH, respectively (Figs. 3A, 4). After sectioning the AENs bilaterally, the total number of Fos‐positive nuclei found after repetitive stimulation of the nasal passages generally decreased (Figs. 3B–D, 4). However, in MDH, there was only a significant decrease in Fos labeling 3 days after AEN sectioning compared with Sham, as indicated by a one‐way ANOVA (*P *= 0.014; Fig. 4B). Additionally, in the paratrigeminal nucleus, there was only a significant decrease in Fos labeling 3 days after AEN sectioning compared with Sham (*P *= 0.02) and Acute (*P *= 0.01), as indicated by *t*‐tests (Fig. 4A). When Fos labeling was plotted at six separate caudal‐rostral levels, a two‐way ANOVA indicated differences between the four groups within the ventral paratrigeminal nucleus (*P *= 0.016; Fig. 5A); post hoc analysis indicated Sham was significantly greater than 3 Day (*P *= 0.015). Within ventral MDH, a significant interaction between groups and caudal‐rostral level precluded main effect analysis using a two‐way ANOVA. Instead, one‐way ANOVAs at individual caudal‐rostral levels indicated significantly more Fos‐positive neurons in Sham at 3 caudal‐rostral levels at and just rostral to calamus scriptorius (*P *= 0.003 at Calamus; *P *= 0.030 at iv; and *P* < 0.001 at v; Fig. 5B).

## Discussion

The present experimental results confirm cardiorespiratory responses to nasal stimulation are attenuated after acute bilateral sectioning of the AENs. The novel findings from these experiments are that the attenuated cardiorespiratory responses to nasal stimulation show a partial restoration after waiting as few as 3 days after bilateral sectioning of the AENs. The bradycardic response to nasal stimulation is significantly more intense 3 days after AEN sectioning compared to Acute AEN sectioning. Immunohostochemical data confirm activation of neurons within MDH and paratrigeminal nucleus after nasal stimulation when the AENs are intact. After the AENs are cut acutely there is a decrease in the number of Fos‐positive neurons within rostral MDH. Surprisingly, 3 days after AEN sectioning the number of Fos‐positive neurons within MDH and paratrigeminal nucleus significantly decreased compared with Sham, even though the cardiorespiratory responses to nasal stimulation intensified. Collectively, these findings indicate, besides the AEN, alternate sensory pathways can activate neurons within the trigeminal nucleus in response to nasal stimulation. The findings further suggest trigeminal neuronal plasticity involving these alternate sensory pathways occurs within 3 days after sectioning of the AENs. Finally, activation of even a significantly reduced number of MDH neurons is sufficient to initiate the nasopharyngeal response.

### Cardiorespiratory responses to nasal stimulation in anesthetized rats

Ammonia stimulation of the nasal passages of rats produced a cardiorespiratory response similarly described by others (Rybka and McCulloch 2006; Panneton et al. 2010b). When both AENs were intact, nasal stimulation caused significant bradycardia, hypertension, and apnea. The bradycardia is parasympathetically mediated, while the increase in MAP is sympathetically mediated through a selective increase in peripheral vasoconstriction (Panneton 2013). In addition, this nasopharyngeal response is similar to that seen during voluntary diving in rats (McCulloch et al. 2010; Panneton et al. 2010a,b, 2012; Chotiyanonta et al. 2013). After acute bilateral sectioning of the AENs, ammonia vapor stimulation of the nasal passages still caused bradycardia, hypertension, and apnea. However, compared to Sham, there was a significantly reduced bradycardia, a significantly increased respiratory rate, and a significantly shorter apnea. The blood pressure response to nasal stimulation was not significantly different between Acute and Sham. These cardiorespiratory responses from rats with acutely sectioned AENs were similar to those described by Rybka and McCulloch (2006). Thus, we confirm the results of Rybka and McCulloch (2006) who concluded the AEN is an important component for the initiation of the nasopharyngeal response in the rat since the response is significantly attenuated after bilaterally cutting the nerves.

In contrast to the results from rats with acutely sectioned AENs, waiting a few days after AEN sectioning produced an intensification of the cardiorespiratory responses to nasal stimulation with ammonia vapors. Waiting 3 or 9 days after AEN sectioning produced a significantly more intense bradycardia compared with Acute. The bradycardia after 3 and 9 day waits were neither significantly different from each other, nor from Sham. Additionally, the respiratory rate and apnea responses in the 3 and 9 day rats were no different from Sham. In contrast, the blood pressure responses to nasal stimulation were not significantly different in the 3 and 9 day rats compared with Acute, or Sham. Thus, the first conclusion from these results is the nasopharyngeal response to nasal stimulation is significantly different 3 days following AEN sectioning versus acute AEN sectioning. A second conclusion is the nasopharyngeal response to nasal stimulation is not significantly different 3 days following AEN sectioning versus when the AENs are intact.

This second conclusion is similar to that reported by Chotiyanonta et al. (2013) who found the cardiorespiratory responses to nasal stimulation with either ammonia vapors or water were identical in rats 9 days after either sham or bilateral sectioning of the AENs. Indeed, it was the discrepancy between the results from Rybka and McCulloch (2006) and Chotiyanonta et al. (2013) that prompted the present investigation. Since the full nasopharyngeal response returned after 9 days (Chotiyanonta et al. 2013), but was attenuated acutely (Rybka and McCulloch 2006), when after AEN sectioning do the nasopharyngeal responses return to those seen in intact animals? Preliminary experiments indicated waiting either 7 or 5 days after AEN sectioning produced responses similar to those found after waiting 9 days. We thus decided to wait just 3 days, and found the cardiorespiratory responses to nasal stimulation are not significantly different compared to when the AENs are intact. Therefore, alternate, non‐AEN, nasal sensory pathways responding to nasal stimulation are only partially functional immediately after AEN sectioning, and become more functional sometime between 0 and 72 h after AEN sectioning.

### Neuronal Fos responses to nasal stimulation in anesthetized rats

The ventral MDH and ventral paratrigeminal nucleus were chosen for Fos quantification in the present study, as these locations show a significant increase in Fos expression during repetitive nasal stimulation with water or ammonia vapors (Dutschmann and Herbert 1997; McCulloch and Panneton 1997; Rybka and McCulloch 2006), or after electrical stimulation of the AEN (Dutschmann and Herbert 1997), compared with unstimulated control animals. Other brainstem locations besides the ventral spinal trigeminal region contained Fos‐positive neurons, but were not quantified because anesthesia can affect the interpretation of Fos expression in efferent areas of a brainstem reflex circuit (McCulloch and Panneton 1997; Coggeshall 2005; Panneton et al. 2012).

As with previous studies, nasal stimulation with ammonia vapors produced an increase in the number of Fos‐positive neurons in both ventral MDH and ventral paratrigeminal nucleus. A limitation to the present study, however, is that we did not use a control group that did not receive any nasal stimulation. This was in effort to reduce the number of research animals used. We feel this is a valid trade‐off, because several previous studies have shown animals not receiving nasal stimulation have a near absence of Fos‐positive neurons in these same brainstem areas (McCulloch and Panneton 1997; McCulloch 2005; Rybka and McCulloch 2006; Panneton et al. 2012; McCulloch et al. 2016).

The central processes of the AEN terminate primarily in the ipsilateral ventral superficial (laminae I and II) MDH and adjacent paratrigeminal nucleus (Panneton et al. 2000, 2006; Hollandsworth et al. 2009). Additionally, the central projections of the AEN co‐localize with neurons activated by nasal stimulation (Hollandsworth et al. 2009). Thus, we expected the observed decrease in Fos‐positive neurons within the ventral MDH in Acute after nasal stimulation, as had been found by Rybka and McCulloch (2006). However, we were surprised there was not a significant difference in the number of Fos‐positive neurons between Sham and Acute in MDH. Since peripheral neural injury can lead to long‐lasting spontaneous activity in sensory neurons (Ambron and Walters 1996), it is possible that the physical act of cutting the AENs secondarily induced MDH and paratrigeminal neurons to express Fos (Rybka and McCulloch 2006). Additionally, there is considerable variability in the time that it takes for Fos to disappear after nerve lesions, with Fos label persisting within the dorsal horn for as long as 14 days (Coggeshall 2005). Further, trigeminal brainstem neurons become hyperexcitable after trigeminal nerve axotomy (Sharp et al. 1989; Terayama et al. 1997; Iwata et al. 2004). Thus, actual sectioning of the AENs may be partially responsible for the number of Fos‐positive neurons not being statistically different in MDH of Sham and Acute rats.

An interesting finding in the 3 day rats was that the number of Fos‐positive neurons within MDH was significantly decreased compared with Sham, even though the cardiorespiratory responses were not significantly different. This suggests relatively few activated neurons are actually necessary to activate the nasopharyngeal response. This conclusion was also made by Panneton et al. (2012), who commented “these [relatively few MDH] neurons invoke a powerful influence over cardiorespiratory behavior with little modification.” These MDH neurons apparently can become activated whether the AENs are intact or sectioned bilaterally.

### Alternate pathways that mediate the nasopharyngeal response in anesthetized rats

After acute bilateral sectioning of the AENs, the cardiorespiratory response to nasal stimulation was attenuated but not completely eliminated. In Acute rats, nasal stimulation still produced decreases in heart rate and respiratory rate and a significant increase in MAP. Thus, after bilaterally sectioning the AENs, nasal stimulation still induced a cardiorespiratory response, albeit an attenuated one. This finding, also found by Rybka and McCulloch (2006), therefore suggests the nasal passages are innervated by nerve(s) other than the AEN, that can by themselves contribute to the activation of brainstem neurons and at least partially induce the nasopharyngeal response. The nasal passages of the rat are innervated by several nerve branches from the ophthalmic and maxillary divisions of the trigeminal nerves (Greene 1963). The nasociliary branch of the ophthalmic division divides into the ethmoidal and internal nasal nerves that innervate the nasal sinus and mucous membrane of the nasal cavity. The pterygopalatine branch of the maxillary division divides into the nasopalatine, posterior superior nasal, and pharyngeal nerves that innervate the nasal cavity, nasal conchae, nasal septum, and nasopharynx. Also, the anterior superior alveolar branch of the maxillary division divides into the nasal nerve that innervates the mucous membrane of the nasal cavity. It is possible any, or all of these nerves, in addition to the AEN, are capable of producing a sensory signal that contributes to the initiation of the nasopharyngeal response. Indeed, it is possible these other nerve(s), in response to nasal stimulation in intact animals, preferentially produce an increase in sympathetic vasomotor activity. The significant increase in MAP during nasal stimulation is identical regardless of whether the AENs are intact or not. In contrast, only attenuated changes in HR and respiration are seen after acute bilateral AEN sectioning. The AEN, on the other hand, can produce all three of the autonomic changes associated with the nasopharyngeal response, as electrical stimulation of the AEN produces bradycardia, hypertension, and apnea (Dutschmann and Herbert 1998; McCulloch et al. 1999; Rozloznik et al. 2009). Thus, only after bilateral sectioning of the AENs can the full contributions of these other afferent nerve(s) be fully revealed.

The present results extend previous findings by demonstrating a restoration of the nasopharyngeal response occurs after waiting as few as 3 days after bilateral sectioning of the AENs. Thus, whatever non‐AEN alternate nerve(s) are involved in providing afferent information that help initiate the nasopharyngeal response, this pathway is only partially functional immediately after AEN sectioning, and is more fully operational within 72 h after nerve sectioning. It seems unlikely it would take a few days for non‐AEN airway receptors to be able to signal the presence of ammonia vapors in the nasal passages. Instead, it is more likely that the restoration of the nasopharyngeal response is due to neuronal plasticity within MDH.

Following nerve axotomy, retrograde signals from the site of injury are sent to the cell bodies to induce axonal repair (Fink and Gainer 1980; Ambron and Walters 1996; Abe and Cavalli 2008). Additionally, signals are sent down *central* branches of these neurons after peripheral nerve injuries (Benowitz et al. 1990), inducing changes within the dorsal horn (Molander et al. 1988; Koerber et al. 1994; Koerber and Mirnics 1996). These changes include both an increase in the number of dorsal horn synapses (Lin et al. 2011) and central sprouting of myelinated afferents (Woolf et al. 1992). However, these dorsal horn changes occur over many weeks, while within the present context, central plasticity within the dorsal horn needs to occur within 3 days to enable the observed restoration of the nasopharyngeal response after AEN sectioning. Additionally, in order for a retrograde signal to play a role in this central plasticity, it must be upregulated at some time point between 0 h and 3 days following the cutting of the AENs. This has led to the labeling of many genes as “regeneration associated genes” (RAGs; (Ma and Willis 2015)). Examples are GAP‐43 (Schreyer and Skene 1993), ATF‐3 (Seijffers et al. 2007), and STAT3 (Schweizer et al. 2002; Qiu et al. 2005). Following nerve injury, significant changes in gene expression are visualized within 8–12 h (Michaelevski et al. 2010). For instance, gene expression of ATF3, JUN, SMAD1, ELF3, and STAT4 is maximal 9 h after sciatic nerve transection (Li et al. 2015). Thus, changes in gene transcription occurring in the trigeminal ganglion (Galef 1982), the site of AEN cell bodies, could provide the neuronal signal helping induce restoration of the nasopharyngeal response after AEN transection.

In addition to any retrograde signal originating in the trigeminal ganglion, central plasticity also needs to occur within 3 days to enable the observed restoration of the nasopharyngeal response after AEN sectioning. After bilateral AEN sectioning, secondary neurons within the MDH receiving afferent input from the AEN may now receive afferent input from other nerves that innervate the nasal passages (see above). This might require establishment and formation of new synapses. Additionally, Chotiyanonta et al. (2013) suggested the existence of previously existing but functionally weak (i.e., “silent”) synapses between non‐AEN nerve(s) and secondary neurons that strengthen and increase their efficacy after AEN sectioning. However, for either of these 2 mechanisms to occur, there must be molecular signaling occurring within the trigeminal nucleus initiating these changes.

Astrocytic glial cells modulate neuronal function and are involved in the establishment and rebuilding of neuronal circuits (Eto et al. 2018). During development, they release various synaptogenic molecules, such as thrombospondins (TSPs), that generate new synapses on dendrites of cortical neurons (Eto et al. 2018). In the spinal dorsal horn, astrocytes activated by peripheral nerve injury contribute to induction of spinal neuronal hyperactivity by releasing various molecules, such as cytokines and chemokines (Gao and Ji 2010). Additionally, within the brainstem spinal trigeminal nucleus, hyperactive astroglia are involved in enhancing neuroplastic responses of MDH neurons following nerve transection (Piao et al. 2006; Okada‐Ogawa et al. 2009; Lo et al. 2011), or experimental tooth movement (Luo et al. 2014). Thus, astrocytic‐mediated synaptogenesis within the MDH could contribute to restoration of the nasopharyngeal response following AEN transection.

## Conclusion

The present experiments were designed to investigate restoration of the nasopharyngeal response after bilateral sectioning of the AENs. Results show after waiting as few as 3 days after bilateral sectioning of the AENs the attenuated cardiorespiratory responses to nasal stimulation show a partial restoration. The bradycardic response to nasal stimulation is significantly more intense 3 days after AEN sectioning compared to immediately after AEN sectioning. Additionally, immunohostochemical data indicate activation of neurons within MDH and paratrigeminal neurons after nasal stimulation both when the AENs are intact and after bilateral sectioning. These findings indicate other nerves innervate the nasal passages, and in the absence of the AEN, can activate neurons within the trigeminal nucleus in response to nasal stimulation. Since there was an attenuation of the cardiorespiratory responses acutely, neuronal plasticity within the trigeminal nucleus involving these alternate sensory pathways require a few days to enable restoration of the nasopharyngeal response.

## Conflict of Interest

Authors have no conflicts of interest to disclose.

## References

[phy213830-bib-0001] Abe, N. , and V. Cavalli . 2008 Nerve injury signaling. Curr. Opin. Neurobiol. 18:276–283. 18655834 10.1016/j.conb.2008.06.005 PMC2633416

[phy213830-bib-0002] Ambron, R. T. , and E. T. Walters . 1996 Priming events and retrograde injury signals. Mol. Neurobiol. 13:61–79. 8892336 10.1007/BF02740752

[phy213830-bib-0003] Anton, F. , and P. Peppel . 1991 Central projections of trigeminal primary afferents innervating the nasal mucosa: a horseradish peroxidase study in the rat. Neuroscience 41:617–628. 1714553 10.1016/0306-4522(91)90354-q

[phy213830-bib-0004] Benowitz, L.I. , N.I., Perrone‐Bizzozero , R.L., Neve , and W., Rodriguez . 1990 GAP‐43 as a marker for structural plasticity in the mature CNS, Progress in brain research. Pp. 309–320,Vol. 86 Elsevier, New York. 2150888 10.1016/s0079-6123(08)63187-8

[phy213830-bib-0005] Butler, P. J. , and D. R. Jones . 1997 Physiology of diving birds and mammals. Physiol. Rev. 77:837–899. 9234967 10.1152/physrev.1997.77.3.837

[phy213830-bib-0006] Chotiyanonta, J. S. , K. M. DiNovo , and P. F. McCulloch . 2013 Bilateral sectioning of the anterior ethmoidal nerves does not eliminate the diving response in voluntarily diving rats. Physiol. Rep. 1:e00141. 24400143 10.1002/phy2.141 PMC3871456

[phy213830-bib-0007] Coggeshall, R. E. 2005 Fos, nociception and the dorsal horn. Prog. Neurobiol. 77:299–352. 16356622 10.1016/j.pneurobio.2005.11.002

[phy213830-bib-0008] Dragunow, M. , and R. Faull . 1989 The use of c‐fos as a metabolic marker in neuronal pathway tracing. J. Neurosci. Methods 29:261–265. 2507830 10.1016/0165-0270(89)90150-7

[phy213830-bib-0009] Dutschmann, M. , and H. Herbert . 1997 Fos expression in the rat parabrachial and Kölliker‐Fuse nuclei after electrical stimulation of the trigeminal ethmoidal nerve and water stimulation of the nasal mucosa. Exp. Brain Res. 117:97–110. 9386008 10.1007/s002210050203

[phy213830-bib-0010] Dutschmann, M. , and H. Herbert . 1998 NMDA and GABAA receptors in the rat Kolliker‐Fuse area control cardiorespiratory responses evoked by trigeminal ethmoidal nerve stimulation. J. Physiol. 510:793–804. 9660894 10.1111/j.1469-7793.1998.793bj.x PMC2231078

[phy213830-bib-0011] Eto, K. , S. K. Kim , I. Takeda , and J. Nabekura . 2018 The roles of cortical astrocytes in chronic pain and other brain pathologies. Neurosci. Res. 126:3–8. 28870605 10.1016/j.neures.2017.08.009

[phy213830-bib-0012] Fink, D. J. , and H. Gainer . 1980 Retrograde axonal transport of endogenous proteins in sciatic nerve demonstrated by covalent labeling in vivo. Science 208:303–305. 6154312 10.1126/science.6154312

[phy213830-bib-0013] Galef, B. G. Jr . 1982 Studies of social learning in Norway rats: a brief review. Dev. Psychobiol. 15:279–295. 7049794 10.1002/dev.420150402

[phy213830-bib-0014] Gao, Y.‐J. , and R.‐R. Ji . 2010 Targeting astrocyte signaling for chronic pain. Neurotherapeutics 7:482–493. 20880510 10.1016/j.nurt.2010.05.016 PMC2950097

[phy213830-bib-0015] Greene, E.C . 1963 Anatomy of the Rat. Hafner: New York.

[phy213830-bib-0016] Hollandsworth, M. P. , K. M. DiNovo , and P. F. McCulloch . 2009 Unmyelinated fibers of the anterior ethmoidal nerve in the rat co‐localize with neurons in the medullary dorsal horn and ventrolateral medulla activated by nasal stimulation. Brain Res. 1298:131–144. 19732757 10.1016/j.brainres.2009.08.077 PMC2760627

[phy213830-bib-0017] Hughes, P. , and M. Dragunow . 1995 Induction of immediate‐early genes and the control of neurotransmitter‐regulated gene expression within the nervous system. Pharmacol. Rev. 47:133–178. 7784478

[phy213830-bib-0018] Iwata, K. , Y. Tsuboi , A. Shima , T. Harada , K. Ren , K. Kanda , et al. 2004 Central neuronal changes after nerve injury: neuroplastic influences of injury and aging. J. Orofac. Pain. 18:293–298. 15636011

[phy213830-bib-0019] Koerber, H. R. , and K. Mirnics . 1996 Plasticity of dorsal horn cell receptive fields after peripheral nerve regeneration. J. Neurophysiol. 75:2255–2267. 8793739 10.1152/jn.1996.75.6.2255

[phy213830-bib-0020] Koerber, H. , K. Mirnics , P. Brown , and L. Mendell . 1994 Central sprouting and functional plasticity of regenerated primary afferents. J. Neurosci. 14:3655–3671. 8207480 10.1523/JNEUROSCI.14-06-03655.1994 PMC6576924

[phy213830-bib-0021] Li, S. , C. Xue , Y. Yuan , R. Zhang , Y. Wang , Y. Wang , et al. 2015 The transcriptional landscape of dorsal root ganglia after sciatic nerve transection. Sci. Rep. 5:16888. 26576491 10.1038/srep16888 PMC4649668

[phy213830-bib-0022] Lin, J.‐Y. , B. Peng , Z.‐W. Yang , and S. Min . 2011 Number of synapses increased in the rat spinal dorsal horn after sciatic nerve transection: a stereological study. Brain Res. Bull. 84:430–433. 21272619 10.1016/j.brainresbull.2011.01.007

[phy213830-bib-0023] Lo, F.‐S. , S. Zhao , and R. S. Erzurumlu . 2011 Astrocytes promote peripheral nerve injury‐induced reactive synaptogenesis in the neonatal CNS. J. Neurophysiol. 106:2876–2887. 21900512 10.1152/jn.00312.2011 PMC3234085

[phy213830-bib-0024] Luo, W. , R. Fu , Y. Tan , B. Fang , and Z. Yang . 2014 Chemokine CCL2 up‐regulated in the medullary dorsal horn astrocytes contributes to nocifensive behaviors induced by experimental tooth movement. Eur. J. Oral Sci. 122:27–35. 24206110 10.1111/eos.12099

[phy213830-bib-0025] Ma, T. C. , and D. E. Willis . 2015 What makes a RAG regeneration associated? Front. Mol. Neurosci. 8:43. 26300725 10.3389/fnmol.2015.00043 PMC4528284

[phy213830-bib-0026] McCulloch, P. F. 2005 Activation of the trigeminal medullary dorsal horn during voluntary diving in rats. Brain Res. 1051:194–198. 15978555 10.1016/j.brainres.2005.05.059

[phy213830-bib-0027] McCulloch, P. F. 2012 Animal models for investigating the central control of the mammalian diving response. Front. Physiol. 3:1–16. 22661956 10.3389/fphys.2012.00169 PMC3362090

[phy213830-bib-0028] McCulloch, P. F. , and W. M. Panneton . 1997 FOS immunohistochemical determination of brainstem neuronal activation in the muskrat after nasal stimulation. Neuroscience 78:913–925. 9153669 10.1016/s0306-4522(96)00633-1

[phy213830-bib-0029] McCulloch, P. F. , K. M. Faber , and W. M. Panneton . 1999 Electrical stimulation of the anterior ethmoidal nerve produces the diving response. Brain Res. 830:24–31. 10350556 10.1016/s0006-8993(99)01374-8

[phy213830-bib-0030] McCulloch, P. F. , K. M. DiNovo , and T. M. Connolly . 2010 The cardiovascular and endocrine responses to voluntary and forced diving in trained and untrained rats. Am. J. Physiol. Regul. Integr. Comp. Physiol. 298:R224–R234. 19923359 10.1152/ajpregu.00592.2009 PMC2806205

[phy213830-bib-0031] McCulloch, P. F. , E. A. Warren , and K. M. DiNovo . 2016 Repetitive diving in trained rats still increases fos production in brainstem neurons after bilateral sectioning of the anterior ethmoidal nerve. Front. Physiol. 7:148. 27148082 10.3389/fphys.2016.00148 PMC4838619

[phy213830-bib-0032] Michaelevski, I. , Y. Segal‐Ruder , M. Rozenbaum , K. F. Medzihradszky , O. Shalem , G. Coppola , et al. 2010 Signaling to transcription networks in the neuronal retrograde injury response. Sci. Signal. 3: ra53. 20628157 10.1126/scisignal.2000952 PMC3645873

[phy213830-bib-0033] Molander, C. , E. Kinnman , and H. Aldskogius . 1988 Expansion of spinal cord primary sensory afferent projection following combined sciatic nerve resection and saphenous nerve crush: a horseradish peroxidase study in the adult rat. J. Comp. Neurol. 276:436–441. 3192769 10.1002/cne.902760308

[phy213830-bib-0034] Okada‐Ogawa, A. , I. Suzuki , B. J. Sessle , C.‐Y. Chiang , M. W. Salter , J. O. Dostrovsky , et al. 2009 Astroglia in medullary dorsal horn (trigeminal spinal subnucleus caudalis) are involved in trigeminal neuropathic pain mechanisms. J. Neurosci. 29:11161–11171. 19741123 10.1523/JNEUROSCI.3365-09.2009 PMC2804401

[phy213830-bib-0035] Panneton, W. M. 2013 The mammalian diving response: an enigmatic reflex to preserve life? Physiology 28:284–297. 23997188 10.1152/physiol.00020.2013 PMC3768097

[phy213830-bib-0036] Panneton, W. M. , P. F. McCulloch , and W. Sun . 2000 Trigemino‐autonomic connections in the muskrat: the neural substrate for the diving response. Brain Res. 874:48–65. 10936223 10.1016/s0006-8993(00)02549-x

[phy213830-bib-0037] Panneton, W. M. , Q. Gan , and R. Juric . 2006 Brainstem projections from recipient zones of the anterior ethmoidal nerve in the medullary dorsal horn. Neuroscience 141:889–906. 16753263 10.1016/j.neuroscience.2006.04.055

[phy213830-bib-0038] Panneton, W. M. , Q. Gan , and T. E. Dahms . 2010a Cardiorespiratory and neural consequences of rats brought past their aerobic dive limit. J. Appl. Physiol. 109:1256–1269. 20705947 10.1152/japplphysiol.00110.2010 PMC2971699

[phy213830-bib-0039] Panneton, W. M. , Q. Gan , and R. Juric . 2010b The rat: a laboratory model for studies of the diving response. J. Appl. Physiol. 108:811–820. 20093670 10.1152/japplphysiol.00600.2009 PMC2853196

[phy213830-bib-0040] Panneton, W. M. , Q. Gan , J. Le , R. S. Livergood , P. Clerc , and R. Juric . 2012 Activation of brainstem neurons by underwater diving in the rat. Front. Physiol. 3:1–13. 22563319 10.3389/fphys.2012.00111 PMC3342523

[phy213830-bib-0041] Paxinos, G. , and C. Watson . 1998 The rat brain in stereotaxic coordinates, 4th ed Academic Press, New York.

[phy213830-bib-0042] Piao, Z. G. , I. H. Cho , C. K. Park , J. P. Hong , S. Y. Choi , S. J. Lee , et al. 2006 Activation of glia and microglial p38 MAPK in medullary dorsal horn contributes to tactile hypersensitivity following trigeminal sensory nerve injury. Pain 121:219–231. 16495005 10.1016/j.pain.2005.12.023

[phy213830-bib-0043] Qiu, J. , W. B. Cafferty , S. B. McMahon , and S. W. Thompson . 2005 Conditioning injury‐induced spinal axon regeneration requires signal transducer and activator of transcription 3 activation. J. Neurosci. 25:1645–1653. 15716400 10.1523/JNEUROSCI.3269-04.2005 PMC6725934

[phy213830-bib-0044] Rozloznik, M. , J. F. R. Paton , and M. Dutschmann . 2009 Repetitive paired stimulation of nasotrigeminal and peripheral chemoreceptor afferents cause progressive potentiation of the diving bradycardia. Am. J. Physiol. 296:R80–R87. 10.1152/ajpregu.00806.2007 18987289

[phy213830-bib-0045] Rybka, E. J. , and P. F. McCulloch . 2006 The anterior ethmoidal nerve is necessary for the initiation of the nasopharyngeal response in the rat. Brain Res. 1075:122–132. 16466647 10.1016/j.brainres.2005.12.112

[phy213830-bib-0046] Schreyer, D. J. , and J. Skene . 1993 Injury‐associated induction of GAP‐43 expression displays axon branch specificity in rat dorsal root ganglion neurons. Developmental Neurobiol. 24:959–970. 10.1002/neu.480240709 8228973

[phy213830-bib-0047] Schweizer, U. , J. Gunnersen , C. Karch , S. Wiese , B. Holtmann , K. Takeda , et al. 2002 Conditional gene ablation of Stat3 reveals differential signaling requirements for survival of motoneurons during development and after nerve injury in the adult. J. Cell Biol. 156:287–298. 11807093 10.1083/jcb.200107009 PMC2199226

[phy213830-bib-0048] Seijffers, R. , C. D. Mills , and C. J. Woolf . 2007 ATF3 increases the intrinsic growth state of DRG neurons to enhance peripheral nerve regeneration. J. Neurosci. 27:7911–7920. 17652582 10.1523/JNEUROSCI.5313-06.2007 PMC6672733

[phy213830-bib-0049] Sharp, F. R. , J. Griffith , M. F. Gonzalez , and S. M. Sagar . 1989 Trigeminal nerve section induces Fos‐like immunoreactivity (FLI) in brainstem and decreases FLI in sensory cortex. Brain Res. Mol. Brain Res. 6:217–220. 2515410 10.1016/0169-328x(89)90057-0

[phy213830-bib-0050] Terayama, R. , N. Nagamatsu , T. Ikeda , T. Nakamura , O. I. Rahman , S. Sakoda , et al. 1997 Differential expression of Fos protein after transection of the rat infraorbital nerve in the trigeminal nucleus caudalis. Brain Res. 768:135–146. 9369310 10.1016/s0006-8993(97)00633-1

[phy213830-bib-0051] Woolf, C. J. , P. Shortland , and R. E. Coggeshall . 1992 Peripheral nerve injury triggers central sprouting of myelinated afferents. Nature 355:75. 1370574 10.1038/355075a0

